# Giant Brunner's Gland Adenoma Presenting as Upper Gastrointestinal Bleeding in 76 Years Old Male: A Case Report

**DOI:** 10.31729/jnma.4013

**Published:** 2019-02-28

**Authors:** Ramesh Rana, Rikesh Sapkota, KC Bishal, Anish Hirachan, Bimas Limbu

**Affiliations:** 1 Department of Internal Medicine, Gautam Buddha Community Heart Hospital, Butwal, Nepal; 2 Department of Cardiology, Gautam Buddha Community Heart Hospital, Butwal, Nepal; 3 Department of Surgery, Manipal Teaching Hospital, Pokhara, Nepal

**Keywords:** *Brunner's gland adenoma*, *Brunner's gland hamartoma*, *Brunner's gland hyperplasia*, *case report*

## Abstract

Brunner's gland adenoma is a rare benign tumor of small bowel, often incidentally discovered during endoscopy or radiological imaging. Mostly they are asymptomatic or often present with nonspecific symptoms such as nausea, vomiting, gastrointestinal hemorrhage, iron deficiency anemia. We reported a 76 years old male case presented with chief complaints of vomiting and black tarry stool. General physical examination was normal except mild tenderness over epigastrium. Esophagogastroduodenoscopy revealed a pedunculated polypoid tubular structure with blind end distally of length approximately 10-12* 3.5*1.5 cm in the second section of the duodenum with multiple skipped ulcers on the exposed surface of it. Additionally, there were few erosions in the duodenum proximally and multiple superficial ulcerations in the antrum, associated with helicobacter pylori confirmed by rapid urease test kit. Surgical or endoscopic excision is the treatment of choice. We consider our case is the eldest case among Brunner's gland adenoma case in literature.

## INTRODUCTION

Brunner's glands adenoma (BGA) is an extremely rare benign variant of small bowel neoplasm arising from the Brunner's gland of the duodenum. Brunner's glands account 10% of benign neoplasm of the duodenum, the first described by Brunner in 1688^1^ whereas Curveilhier described first case in 1835.^2,3^ The pathophysiology of this is unknown; however, it is believed chronic insult of Brunner's gland secondary to H. pylori infection would contribute to its hyperplasia.

Generally, it is incidentally diagnosed during upper gastrointestinal endoscopy or sometimes computed tomography and magnetic resonance imaging as a tubular or cystic lesion in the duodenum.^2,4^ Mostly they are asymptomatic or present with chronic abdominal pain, nausea, vomiting, gastrointestinal bleedings, and anemia.^5^

We are reporting a rare case and eldest case of BGA presented with upper gastrointestinal bleeding.

## CASE REPORT

**Figure 1.**
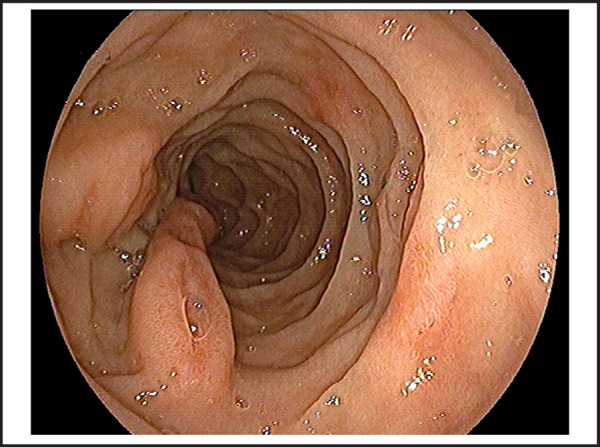
Brunner's gland adenoma with multiple ulcers on surface initial view with major papillary opening.

**Figure 2.**
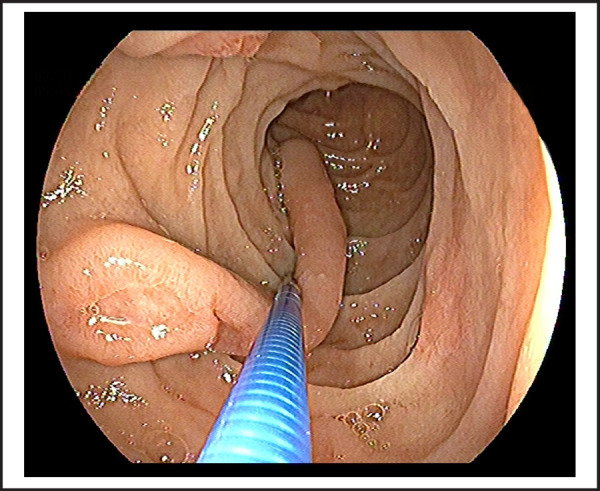
Brunner's gland adenoma visualizing blind end with the help of biopsy forcep.

**Figure 3.**
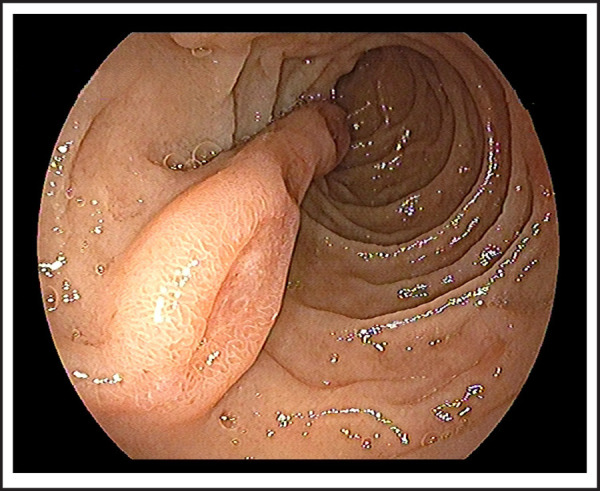
Brunner's gland adenoma with base and multiple skipped ulcers on exposed surface.

76 years old male patient visited on the outpatient department with chief complaints of vomiting of blackish color contents and melena for 2 days. He had chronic episodic upper central abdominal pain for many years subsided by antisecretory medications; however, no history of melena previously. There was no significant previous medical and surgical history or investigation done such as endoscopy. On presentation, he was hemodynamically stable with normal vitals and biochemistry and hemoglobin was 11.9 gm/dl; however, stool for occult blood was positive and black tarry stool on per rectal examination. Therefore, he was admitted and pantoprazole 80 mg stat, then followed by 8mg/ hr via infusion pump given along with oral sucralfate. We performed an esophagogastroduodenoscopy on day second of admission, showed pedunculated and elongated tubular blind end distally polypoid mass with an approximate length of~10-12 cm, broad base 3.5*1.5 cm size, arising about the same level of the major papillary opening in second part of the duodenum (Figure 1 and Figure 2). There were multiple skipped eroding ulcers noted on the same exposed surface of polypoid mass (Figure 3); however, no recent bleeding stigmata noted. Additionally, few erosions and an ulcer noted on opposite surface proximal to it including duodenal bulb and multiple erosions with superficial ulcers noted in the antrum without stigmata of bleeding which was associated with Helicobacter pylori infection, revealed by rapid urease test. He was managed conservatively in our center, then after referred to another center for endoscopic excision of the polypoid mass.

## DISCUSSION

Brunner's gland adenoma or Brunneroma is an extremely rare benign small bowel tumor arising from the Brunner's gland commonly in the second part of the duodenum. It is also known as Brunner's gland hamartoma. Although it is a rare variant with the incidence of <1 percent, there are many cases reports published in the literature.^6^ The exact pathophysiology of the Brunner's gland adenoma remains obscure, however, there are many postulations regarding the etiology of BGH. Increased gastric acid secretion is believed to be the cause of Brunner's gland hyperplasia as Brunner's gland maintains the alkaline condition in the duodenum, and also an association of BGH and achlorhydria; but Spellberg et al. didn't found regression of lesion with anti-acid secretary medication. Furthermore, Helicobacter pylori infection may play a major role in Brunner's gland hyperplasia or adenoma. It has been reported that there was the association of HP infection and BGA in 71% of cases,^7^ however, there was no HP association in both cases in another case report. ^6^ Above all, the pathogenesis remains duodenal dysembryoplastic lesion or hamartoma.

Generally, Brunner's gland adenoma is asymptomatic or presents with nonspecific symptoms with abdominal discomfort, nausea, vomiting, gastrointestinal bleeding, intussusception, or iron deficiency anemia. Recent studies reported melena as the presenting complaints as in our case although he had chronic episodic abdominal discomfort for 2 years.^8,9^ Those who presented with melena had larger size with maximum size reported up to 12cm,^5^ in our case also length was approximately 10-12 cm which is longer as previously reported.^9^ Additionally, age of the patient was older (76 years) than previously reported of 73 years old.^1^

Brunner's gland adenoma commonly presented as hyperplastic or hamartomatous mucosal changes are always benign; however, rarely foci of metaplasia to malignant foci have been reported.^10^ According to a recent study, dysplastic changes noted in 2.1% and invasive carcinoma in 0.3% of all Brunner's gland hyperplasia.^2,10^ Surgical resection is the gold standard especially for giant Brunner's gland hamartoma and unresectable mass; however, recent studies have prevailed endosco-pic resection of the large adenoma also have been safely done without complications. ^5^

In conclusion, although Brunner's gland adenoma have a very low incidence, recently more cases are reported in the literature, they can present as gastrointestinal bleeding, and diagnosed by endoscopic or radiological imaging. Surgical resection or endoscopic resection is equally possible in recent days.
